# Histone Lysine Methyltransferase SETD2 Regulates Coronary Vascular Development in Embryonic Mouse Hearts

**DOI:** 10.3389/fcell.2021.651655

**Published:** 2021-04-09

**Authors:** Fengling Chen, Jiewen Chen, Hong Wang, Huayuan Tang, Lei Huang, Shijia Wang, Xinru Wang, Xi Fang, Jie Liu, Li Li, Kunfu Ouyang, Zhen Han

**Affiliations:** ^1^Department of Cardiovascular Surgery, Peking University Shenzhen Hospital, State Key Laboratory of Chemical Oncogenomics, School of Chemical Biology and Biotechnology, Peking University Shenzhen Graduate School, Shenzhen, China; ^2^Department of Medicine, University of California, San Diego, La Jolla, CA, United States; ^3^Department of Pathophysiology, School of Medicine, Shenzhen University, Shenzhen, China; ^4^State Key Laboratory of Oncogenes and Related Genes, Renji-Med X Clinical Stem Cell Research Center, Ren Ji Hospital, School of Medicine and Biomedical Engineering, Shanghai Jiao Tong University, Shanghai, China; ^5^School of Biomedical Engineering and Med-X Research Institute, Shanghai Jiao Tong University, Shanghai, China

**Keywords:** SETD2, H3K36me3, cardiac development, coronary vessel development, embryonic development

## Abstract

Congenital heart defects are the most common birth defect and have a clear genetic component, yet genomic structural variations or gene mutations account for only a third of the cases. Epigenomic dynamics during human heart organogenesis thus may play a critical role in regulating heart development. However, it is unclear how histone mark H3K36me3 acts on heart development. Here we report that histone-lysine N-methyltransferase SETD2, an H3K36me3 methyltransferase, is a crucial regulator of the mouse heart epigenome. *Setd2* is highly expressed in embryonic stages and accounts for a predominate role of H3K36me3 in the heart. Loss of *Setd2* in cardiac progenitors results in obvious coronary vascular defects and ventricular non-compaction, leading to fetus lethality in mid-gestation, without affecting peripheral blood vessel, yolk sac, and placenta formation. Furthermore, deletion of *Setd2* dramatically decreased H3K36me3 level and impacted the transcriptional landscape of key cardiac-related genes, including *Rspo3* and *Flrt2*. Taken together, our results strongly suggest that SETD2 plays a primary role in H3K36me3 and is critical for coronary vascular formation and heart development in mice.

## Introduction

The mammalian heart is the first functional organ and the first indicator of life, and its normal formation and function are essential for fetal life (Bruneau, 2013). This complex process of heart development comprises of cardiomyocyte differentiation, cardiac organogenesis, coronary vessel formation, and excitation–contraction coupling, intricately regulated by a group of core cardiac transcription factors in a coordinated temporal and spatial manner (Martinez et al., 2015; Hota and Bruneau, 2016; Cui et al., 2018). Congenital heart defect (CHD) is the leading cause for birth defects, perinatal death, and death in children worldwide (Zhang and Liu, 2015). However, the causes of CHD are not yet completely understood; most types of CHD belong to complex genetic diseases, which are caused by the interaction between genetic factors and environmental factors (Kalisch-Smith et al., 2020). Recent studies have shown that epigenetic regulation, particularly histone lysine methylation modification, may be involved in the development of heart and blood vessels and responsible for the etiology of CHD progression (Yi et al., 2017). Posttranslational methylation of Histone 3 lysine 36 (H3K36me) is an important epigenetic marker that contributes to the functionality of the chromatin. Histone lysines can be methylated in different forms: mono- (me1), di− (me2), or trimethylated (me3); the balance is precisely controlled by the lysine methyltransferases and the lysine demethylases (KDMs) (Black and Whetstine, 2013; Hyun et al., 2017). It has been reported that haploinsufficiency of H3K36me2 methyltransferase NSD1 causes Sotos syndrome with congenital heart defects (Kurotaki et al., 2002). Moreover, deletion of NSD2 also causes Wolf–Hirschhorn syndrome, characterized by growth retardation and congenital cardiovascular anomalies (Nimura et al., 2009). In addition, JMJD5, another H3K36me2 histone demethylase that is highly expressed in the heart, also plays an essential role in regulating cardiac and embryonic development (Ishimura et al., 2012; Oh and Janknecht, 2012). All these studies clearly highlighted the role of H3K36me2 in cardiac development. However, the physiological function of H3K36me3 in cardiac development remains largely unclear.

H3K36me3 has been shown to be involved in transcription elongation, pre-mRNA splicing, DNA methylation, and DNA damage repair (McDaniel and Strahl, 2017). The SET domain-containing protein SETD2 has been recognized as the predominant methyltransferase in mammals that can tri-methylate histone H3 at lysine 36 (Sun et al., 2005). Furthermore, previous studies using genetically engineered mouse models have suggested that SETD2 may regulate multiple physiological processes including spermiogenesis (Zuo et al., 2018), oocyte development (Li et al., 2018), V(D)J recombination in lymphocytes (Ji et al., 2019; Chu et al., 2020), and antiviral immunity (Chen et al., 2017). SETD2 may also play an important role in embryonic stem cells (Zhang et al., 2014), hematopoietic stem cells (Zhang et al., 2018; Zhou et al., 2018), and bone marrow mesenchymal stem cells (Wang et al., 2018). Notably, global SETD2 knockout mice are embryonically lethal at around E10.5, developing severe defects in blood vessel development (Hu et al., 2010), suggesting that SETD2 might play a role in regulating cardiovascular development. However, the cell type-specific mechanism and how SETD2 regulates cardiac development remain unknown.

To investigate the role of SETD2 and its related H3K36me3 in cardiac development, we generated a mouse model with deletion of the *Setd2* gene by Mesp1-Cre that targets multiple cardiovascular lineages including cardiomyocytes, vascular endothelial cells, and endocardial cells. We demonstrated that deletion of SETD2 dramatically decreased the level of H3K36me3 but not H3K36me1 and H3K36me2 in embryonic hearts. Furthermore, we found that SETD2 deficiency dramatically impaired coronary vascular formation and ventricular non-compaction and eventually resulted in embryonic lethality. We also performed chromatin immunoprecipitation sequencing (ChIP-seq) and mRNA sequencing (mRNA-seq) and demonstrated that some key cardiac-related genes including *Rspo3* and *Flrt2* could be downregulated by SETD2 deletion during cardiac development. All these results demonstrated that SETD2 is a key regulator of H3K36me3 in embryonic hearts and plays a critical role in cardiac development by regulating coronary vessel formation.

## Materials and Methods

### Mice

All mouse lines have been previously described: *Setd2-floxed* alleles (Wang et al., 2018; Zuo et al., 2018) and *Mesp1-Cre^+^* mouse (Saga et al., 1999, 2000). All mouse lines were of mixed C57BL6/ICR background. To generate cardiac precursor-specific *Setd2* knockout mice, *Setd2*^*f/f*^ mice were first bred with *Mesp1-Cre^+^* mice to generate *Mesp1-Cre^+^Setd2*^*f/*+^ mice, which were further crossed with *Setd2*^*f/f*^ mice to generate *Mesp1-Cre^+^Setd2*^*f/f*^ (mKO) mice, and the littermate *Mesp1-Cre*^–^*Setd2*^*f/*+^ and *Mesp1-Cre*^–^*Setd2*^*f/f*^ mice were used as control.

### DNA Analysis

Genomic DNA was extracted from mouse tails or embryonic yolk sac as previously described (Duan et al., 2019), and polymerase chain reaction (PCR) was used to characterize the genotypes of the offspring using the following gene-specific primers (from 5′ to 3′): *Setd2* (forward, GTAAAGTAGTATTATGCCAAGGCCC; reverse, TATTTAAACTCTCTCTGGGGGTGG), *Mesp1-Cre* (forward, CTCTGAGCATGGTTCTTTCAAC; reverse, TCCCTGAACATGTCCATCAGGTTC).

### Quantitative Real-Time PCR

RNA samples were prepared, and quantitative real-time PCR (qRT-PCR) was performed as previously described (Lin et al., 2016). Briefly, RNA was extracted using RNeasy minikit (Qiagen), and cDNA was synthesized using *TransScript* One-Step gDNA Removal and cDNA Synthesis SuperMix Kit (Transgen Biotech). qRT-PCR was performed using *TransScript* Tip Green qPCR SuperMix (Transgen Biotech) according to the manufacturer’s instructions, using the gene-specific primers (Supplementary Table 1). Each sample was run at least in duplicate. *Gapdh* was used as the internal control, and relative transcript abundance of each gene was then normalized to *Gadph*.

### Histological Analysis

Embryos were freshly collected in ice-cold phosphate-buffered saline (PBS) after a timed mating using a 12-h light/dark cycle, with noon on the day of discovering a vaginal plug defined as E0.5, and images were captured under stereo microscopes (Zeiss Stemi 2000-C). Tissues were then fixed overnight in 4% paraformaldehyde (PFA) made in PBS, progressively dehydrated in gradient ethanol, and embedded in paraffin. Sections of 5-μm thickness were collected on slides and subjected to hematoxylin and eosin (H&E) staining as previously described (Lin et al., 2019). The thickness of the ventricle and trabecular area was measured and analyzed with ImageJ software.

### mRNA Sequencing (mRNA-Seq) and Data Analysis

Individual embryonic mouse hearts were freshly isolated in cold 0.1% diethyl pyrocarbonate-treated PBS and immediately frozen in liquid nitrogen and then stored at −80°C. Genotypes were examined by PCR on yolk sacs, and three isogenic hearts were then pooled as one sample. Total RNA was extracted using RNeasy Mini Kit (Qiagen), quantified by Agilent 2100 Bioanalyzer (Agilent Technologies, Palo Alto, CA, United States) and NanoDrop (Thermo Fisher Scientific Inc.), and qualified by 1% agarose gel. One microgram of total RNA with a RIN value above 6.5 was used for subsequent library preparation. The poly(A) mRNA isolation was performed using Poly(A) mRNA Magnetic Isolation kit (Vazyme, NR611). mRNA fragmentation and priming were performed using First Strand Synthesis Reaction Buffer and Random Primers. Sequencing was carried out using a 2 × 150-bp paired-end (PE) configuration; image analysis and base calling were conducted by the HiSeq Illumina instrument. The sequencing data were filtered by Cutadapt (V1.9.1) and then aligned to the *mus musculus* genome via software Hisat2 (v2.0.1). Gene expression levels were estimated by HTSeq (v0.6.1). Differential expression analysis used the DESeq2 Bioconductor package, the estimates of dispersion and logarithmic fold changes incorporate data-driven prior distributions, and corrected *P* values (*q*-value) of genes were set ≤ 0.05 to detect differential expressed ones. Gene Ontology (GO) enrichment analysis of differentially expressed genes was implemented by GOseq (v1.34.1), in which gene length bias was corrected. GO terms with a *q*-value ≤ 0.05 were considered significantly enriched for the differentially expressed genes. KEGG (Kyoto Encyclopedia of Genes and Genomes) is a database resource for understanding high-level functions and utilities of biological systems. KOBAS software (KOBAS, Surrey, United Kingdom) was used to test for statistically significant enrichment of differentially expressed genes in KEGG pathways. Data were deposited to the National Center for Biotechnology Information BioSample database (PRJNA692266).

### EdU Assay and Immunostaining

For EdU labeling, dams were injected with EdU (15 mg/kg, Ribobio) that was diluted in PBS 2 h prior to embryo harvesting, collected in ice-cold PBS, and fixed overnight in 4% PFA at 4°C overnight. The tissues were then incubated with an ascending series of sucrose concentrations from 10% to 25% and embedded in optimal cutting temperature (OCT) compound (Sakura Finetek United States Inc., Torrance, CA, United States). Cryosections (10 μm) were prepared, and immunostaining was performed as previously described (Fan et al., 2020). The cell proliferation rate was measured using the Cell-Light EdU Apollo488 *in vitro* Kit (Ribobio, C10310-3) and the immunofluorescence staining using the antibody recognizing phospho-Histone 3 (antibody from Millipore, 2465253). Cell apoptosis was assessed by immunofluorescence staining using the antibody recognizing cleaved-Caspase 3 (Cell Signaling Technologies, 9661). α-Actinin (Sigma, A7811) and PECAM1 (BD Biosciences, 550274) were used to stain cardiac and endothelial cells, respectively. DAPI was used to counterstain nuclei. The slides were imaged and subjected to an independent blinded analysis, using the Olympus IX73 confocal microscope and ImageJ software.

### Whole-Mount PECAM1 Staining of Embryonic Hearts

Whole-mount PECAM1 staining was performed as previously described (Yang et al., 2020). In brief, hearts were dissected from mouse embryos in ice-cold PBS and fixed in 4% PFA at 4 °C overnight. Tissues were blocked in 0.2% Triton 100 solution containing 5% BSA and 2% horse serum and stained with anti-PECAM1 antibody (BD bioscience, 550274; 1:20) overnight at 4°C. Subsequently, the samples were washed and incubated with Alexa-conjugated secondary antibodies (Invitrogen) for 2 h at room temperature. The pictures were acquired with an Olympus IX73 fluorescence microscope.

### Western Blotting

Freshly isolated heart samples were prepared and homogenized in lysis buffer (8 M urea, 2 M thiourea, 75 mM DTT, 3% SDS, 0.03% Bromophenol Blue, 0.05 M Tris–HCl pH 6.8). Standard procedures were used for SDS-PAGE and subsequent transfer to PVDF membranes (Millipore, United States). The primary antibodies include anti-H3K36me1 (Abcam, ab9048), anti-H3K36me2 (Abcam, ab9049), H3K36me3 (Abcam, ab9050), and anti-Histone H3 (Cell Signaling Technologies, 4499). The secondary antibody is HRP-linked anti-rabbit IgG1 (Cell Signaling Technologies, 7074).

### Chromatin Immunoprecipitation Sequencing (ChIP-seq) and ChIP-qPCR

Embryonic mouse hearts were freshly isolated in cold PBS containing Protease Inhibitor Cocktail and immediately frozen in liquid nitrogen, and then stored at −80°C. Eight isogenic hearts were pooled as one sample. ChIP assays were carried out following Chromatin IP kit protocol (Cell Signaling Technologies, 9005). Briefly, tissues were fixed with 1.5% formaldehyde to cross-link histone proteins to DNA and disaggregated using a homogenizer. The nuclei were prepared with mild lysis, and chromatin was digested with micrococcal nuclease into 150–900 bp DNA/protein fragments. Two percent of input samples were retained and stored for further use. Afterward, the antibody recognizing H3K36me3 (Abcam, ab9050) and IgG (Cell Signaling Technologies, 2729) were added, respectively, and the complex co-precipitates were captured by Protein G magnetic beads. Chromatin was eluted from antibody/Protein G magnetic beads, and cross-links were reversed. DNA was purified from all samples including 2% input sample and stored at −20°C, which were ready for following sequencing analysis. ChIP and input samples were quantified using a Qubit 2.0 Fluorometer (Invitrogen, Carlsbad, CA, United States) and qualified by Agilent Bioanalyzer 2100 (Agilent Technologies, Palo Alto, CA, United States). For each sample, at least 10 ng ChIP product was used for library preparation. The ChIP product was treated with End Prep Enzyme Mix for end repairing, 5′ phosphorylation, and dA-tailing in one reaction, followed by ligation to adaptors with a “T” base overhang. Adaptor-ligated DNA was then recovered using AxyPrep Mag PCR Clean-up (Axygen). Each sample was then amplified by PCR for 8 cycles using P5 and P7 primers, with both primers carrying sequences which can anneal with flow cell to perform bridge PCR and P7 primer carrying a six-base index allowing for multiplexing. The PCR products were cleaned up using AxyPrep Mag PCR Clean-up, validated using an Agilent 2100 Bioanalyzer, and quantified by Qubit 2.0 Fluorometer. Then, the libraries with different indexes were multiplexed and loaded on an Illumina instrument according to the manufacturer’s instructions (Illumina, San Diego, CA, United States). Sequencing was carried out using a 2 × 150 paired-end (PE) configuration; image analysis and base calling were conducted by the HiSeq Control Software (HCS) + OLB + GAPipeline−1.9 (Illumina) on the Illumina instrument. Pass-filter data of fastq format were processed by Cutadapt (version 1.9.1) to be high-quality clean data. Firstly, reference genome sequences and gene model annotation files of relative species were downloaded from NCBI. Next, bowtie2-build was used to index the reference genome sequence. Finally, clean data was aligned to the reference genome via software Bowtie2 (version 2.2.6). Input control was used as a reference to analyze peak quality control, peak calling, and peak annotation by MACS (V2). qPCR was used to amplify various regions of the target gene genome, and primers for ChIP-qPCR primers are listed in Supplementary Table 2.

### Statistics

*P* values were calculated using a 2-tailed, unpaired Student’s *t* test, or 2-way ANOVA with Bonferroni post-hoc test for multiple comparisons. Data represent mean ± SEM. *P* < 0.05 was considered statistically significant.

### Ethics Statement

All mice were housed under a 12-h day/night cycle at a temperature of 25°C. All animal care and experiments were conducted in accordance with the guidelines established by the Animal Care and Use Committee (IACUC) at Peking University Shenzhen Graduate School (Shenzhen, China) and approved by the IACUC (Approval #: AP0017). A periodic review of procedures was performed, and amendments were made as needed.

## Results

### Temporal Expression of Setd2 and H3K36me3 in Heart Development

We first investigated the expression of *Setd2* and the levels of H3K36me3 in mouse cardiac tissues at different stages. We collected mouse hearts at embryonic day 9.5 (E9.5), E10.5, E12.5, and E15.5, the postnatal day 1 (P1), P7, P21, and P60 and measured *Setd2* mRNA levels, respectively. We found that *Setd2* was highly expressed at the early stages of heart development, from E9.5 to P7, and then significantly decreased thereon after (Supplementary Figure 1A). Notably, *Setd2* expression is about three to five folds higher at E9.5-P7 compared to P21 or adult heart, which is consistent with the GEO profile from NCBI (GDS5003). Since P7 has been considered as an important turning point for cardiomyocyte maturation (Porrello et al., 2011; O’Meara et al., 2015), our results implicated that *Setd2* might play a predominant role in regulating early cardiac development. Interestingly, in contrast to *Setd2* expression, the levels of H3K36me3 remained relatively stable from E12.5 to adult (Supplementary Figures 1B,C), indicating a potential steady requirement of H3K36me3 in cardiac tissues at both embryonic and adult stages.

### Deletion of Setd2 by Mesp1-Cre Leads to Embryonic Lethality and Abnormal Cardiac Development

Although it has been shown that global *Setd2* knockout mice are embryonic lethal at around E10.5 and exhibit severe vascular abnormalities in the embryo, yolk sac, and placenta (Hu et al., 2010), the cell type-specific mechanism underlying the embryonic lethality of global *Setd2* knockout mice and the role of SETD2 in cardiovascular development have not been well understood. Here, we crossed *Setd2*^*f/f*^ mice with the *Mesp1-Cre* transgenic mice to generated *Mesp1-Cre*^+^*Setd2*^*f/f*^ (mKO) mice (Supplementary Figure 2A). Mesp1, a transcription factor of the b-HLH family, is the earliest marker of the cardiovascular lineages (Saga et al., 1999, 2000). Our and other studies have demonstrated that Mesp1-Cre can target multiple cardiovascular lineages including cardiomyocytes and vascular endothelial cells in the early-developing mouse embryos (Saga et al., 1999; Yang et al., 2020). We then collected hearts from E12.5 and E13.5 embryos and found that the mRNA levels of *Setd2* were dramatically reduced in mKO hearts at both stages when compared with control hearts (Supplementary Figure 2B), suggesting that SETD2 could be efficiently ablated by Mesp1-Cre in mutant hearts. Moreover, H3K36me3 was also significantly decreased in mKO heart at both time points (Supplementary Figures 2C,D), demonstrating that deletion of SETD2 is sufficient to reduce the levels of H3K36me3 in embryonic hearts though additional methyltransferases may also exist.

**FIGURE 1:**
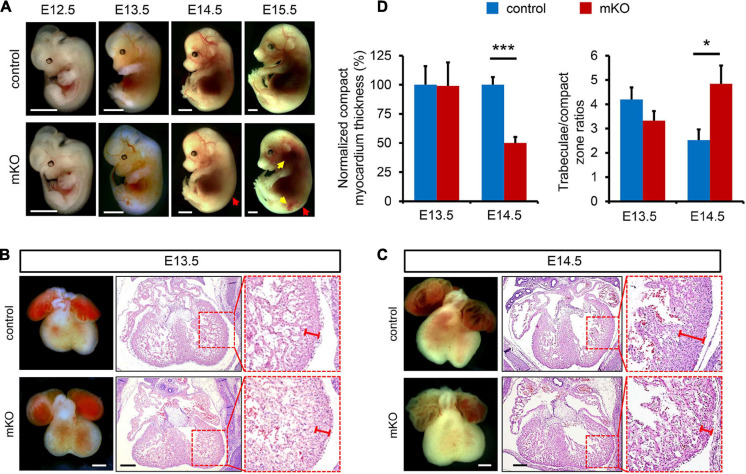
Deletion of SETD2 leads to abnormal cardiac development. **(A)** Representative morphology of control and *Mesp1-Cre*^+^*Setd2*^*f/f*^ (mKO) embryos at E12.5, E13.5, E14.5, and E15.5. Please note that mKO embryos show subcutaneous edema (red arrow) and hemorrhage (yellow arrow) after E14.5. Scale bar, 2 mm. **(B,C)** Morphological and histological analyses of control and mKO hearts at E13.5 **(B)** and E14.5 **(C)**. Boxed areas are magnified on the right. The ventricular compact zone is indicated by red lines. Scale bar, 400 μm. **(D)** Quantitative analysis of the thickness of the myocardium compact zone and the degree of hypertrabeculation at E13.5 (*n* = 5 for each group) and E14.5 (*n* = 4 for each group). The measurements were acquired in three evenly spaced regions along the perimeters of left ventricles. All data represent mean ± SEM. Significance was determined by two-tailed, unpaired Student’s *t* test. *p < 0.05, ***p < 0.001 versus control.

Notably, genotypic analysis revealed that no mKO pups were observed at weaning (Table 1), while the heterozygous *Mesp1-Cre*^+^*Setd2*^*f/*+^ (hKO) offspring and control pups were healthy and fertile. Furthermore, no mKO mice could be observed at P1, suggesting that mKO mice could be embryonically lethal during embryonic development. The mKO embryos could be observed at Mendelian ratios at E16.5, but all mKO embryos observed at this stage were necrotic and partially absorbed (Table 1). In fact, subcutaneous edema and hemorrhage could be found in mKO embryos as early as E14.5, but mKO embryos were phenotypically undistinguishable from the littermate control embryos at E13.5 (Figure 1A).

**TABLE 1:** Genotypic analysis of the offspring from *Mesp1-Cre*^+^ *Setd2*^*f/+*^ × *Setd2*^*f/f*^ intercrosses.

**Age**	***Mesp1-Cre^–^ Setd2^*f/+*^***	***Mesp1-Cre^–^ Setd2^*f/f*^***	***Mesp1-Cre^+^ Setd2^*f/*+^***	***Mesp1-Cre^+^ Setd2^*f/f*^***	**Total**
E12.5	14 (25.9%)	12 (22.2%)	17 (31.5%)	11 (20.4%)	54
E13.5	49 (26.8%)	35 (19.1%)	53 (29.0%)	46 (25.1%)	183
E14.5	19 (20.2%)	26 (27.7%)	19 (20.2%)	30 (31.9%)^a^	94
E15.5	9 (32.1%)	8 (28.6%)	4 (14.3%)	7 (25.0%)^a,b^	28
E16.5	1 (7.7%)	5 (38.5%)	2 (15.4%)	5 (38.5%)^c^	13
P1	22 (36.1%)	22 (36.1%)	17 (27.9%)	0 (0.0%)	61
P21	15 (36.6%)	12 (29.3%)	14 (34.1%)	0 (0.0%)	41

*The embryos or pups were collected from embryonic day 12.5 (E12.5) to postnatal day 1 (P1) for genotyping, respectively. The numbers in brackets represented the percent of the indicated genotype to the total embryos in each corresponding period.*

*^a^All embryos exhibited subcutaneous edema on the back.*

*^b^All embryos exhibited subcutaneous hemorrhage.*

*^c^All embryos are necrotic.*

We further performed more histological analysis to determine the effects of deletion of SETD2 by Mesp1-Cre on cardiac and vascular development. First, we did not observe any apparent difference in embryonic vascular development between control and mKO mice at E11.5, as assessed by whole-mount PECAM1 staining (Supplementary Figure 3A). Histological assessment also indicated that embryonic mKO hearts at E12.5 and E13.5 are largely normal when compared with control hearts (Figures 1B,D and Supplementary Figure 3B). However, mKO hearts looked not developing further after E13.5, indicated by the fact that the appearance of mKO hearts from E14.5 to E16.5 still retained as E13.5 and the lack of a typical triangle apex in the left ventricles appeared in mutant hearts after E13.5 (Figures 1B,C; Supplementary Figures 4A,B). From E14.5, ventricular non-compaction indicated by a significant decrease in the thickness of compact zone was observed in all mKO hearts. Accordingly, a phenotype of hypertrabeculation that has been assessed by the ratio of trabecular area to ventricular compact zone area (Ishiwata et al., 2003) was also observed in mKO hearts at the same stage (Figure 1D). Myocardial compaction has been generally considered as the final phase of cardiac development, in which the majority of heart muscles transform from a sponge-like meshwork to a thick, densely compacted muscle layer (Sedmera et al., 2000; Hussein et al., 2015). Such a defect in myocardial compaction was further exacerbated in mKO hearts at the later stages, indicated by a nearly single layer of cardiomyocytes in the compact zone of mKO ventricles (Supplementary Figures 4A,B). Furthermore, we also observed ventricular septal defect (6 off 7) and double outlet right ventricle (3 off 7) in mKO hearts at E15.5 (Supplementary Figure 4A). All these results together demonstrated that deletion of SETD2 by Mesp1-Cre resulted in embryonic lethality and various cardiac malformation including ventricular non-compaction in mice.

### Coronary Vessel Formation Is Impaired in SETD2-Deficient Hearts

We then investigated the mechanisms underlying the role of SETD2 on myocardial compaction. First, we measured cardiac cell proliferation and apoptosis in control and mKO hearts at E13.5 and E14.5. However, the rates of cardiomyocytes proliferation were comparable between control and mKO hearts at both stages (Supplementary Figures 5A–D). We also did not see obvious cell apoptosis in mKO cardiomyocytes at both stages (Supplementary Figure 5E). These results indicated that loss of SETD2 did not lead to cardiac hypoplasia by reducing proliferation or increasing apoptosis of cardiomyocytes in mutant hearts.

**FIGURE 2:**
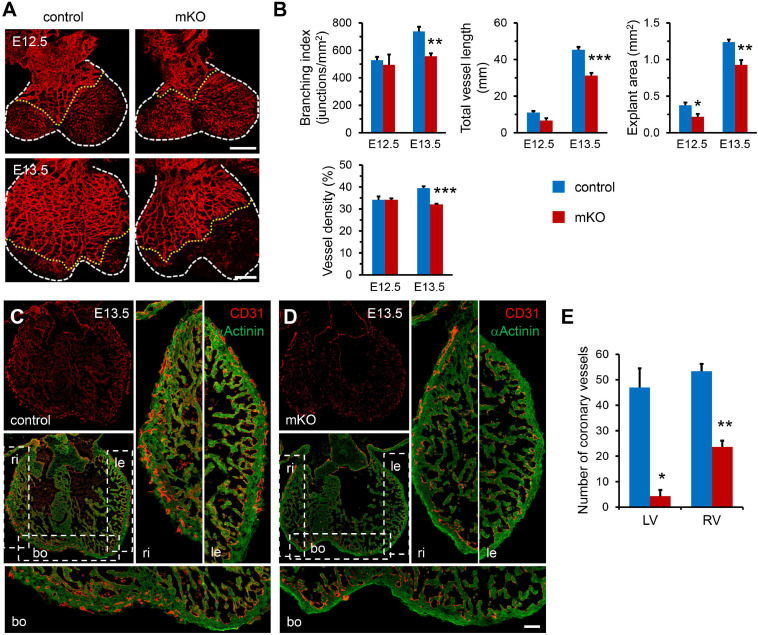
Loss of SETD2 causes defects in coronary vascular development. **(A)** Whole-mount PECAM1 immunostaining of control and mKO embryonic hearts at E12.5 and E13.5. Scale bar, 500 μm. White dotted line indicates the border of heart. Yellow dotted line indicates the leading tips of coronary vessels. **(B)** Quantitative analysis of coronary vessels including branching index, total vessel length, explant area, and vessel density in control and mKO embryonic hearts at E12.5 and E13.5. *n* = 3 for each group at E12.5, *n* = 5 for each group at E13.5. All data represent mean ± SEM. Significance was determined by two-tailed, unpaired Student’s *t* test. *p < 0.05, **p < 0.01, ***p < 0.001 versus control. **(C,D)** Immunostaining analysis of coronary vessel’s distribution in control **(C)** and mKO **(D)** hearts at E13.5. Hearts were sectioned and stained with anti-α-actinin antibody for cardiomyocytes and anti-CD31 antibody for coronary vascular endothelial cells. Scale bar, 40 μm. Ri, right ventricle. Le, left ventricle. Bo, bottom. **(E)** Quantification of the number of coronary vessels in left (LV) and right ventricles (RV) of control and mKO hearts at E13.5, respectively. *n* = 5 for each group. All data represent mean ± SEM. Significance was determined by two-tailed, unpaired Student’s *t* test. *p < 0.05, **p < 0.01 versus control.

On the other hand, formation of a functional coronary vasculature has been shown to promote ventricular myocardium thickening and compaction and thus play a critical role in cardiac development (Zhang et al., 2013; Wu, 2018). Therefore, we next examined whether deletion of SETD2 could influence the development of coronary vessels in mice. In the developing heart, a set of sprouting venous endothelial cells migrate and invade the muscle layer of the heart, forming the nascent coronary vessel plexus at E12.5, and subsequently remodeled into arteries, veins, and capillaries (Red-Horse et al., 2010; Tian et al., 2015; Su et al., 2018). However, the emergence of coronary vessel plexus was basically normal in mKO hearts since both control and mKO hearts at E12.5 possessed coronary vessel stems migrating from sinus venosus without obvious disparity when measured by the whole-mount PECAM1 staining in hearts (Figure 2A). However, deletion of SETD2 significantly reduced the spreading of coronary vessels from the base to the apex in the heart, indicated by the fact that a significant decrease of explant area could be found in mKO hearts as early as E12.5 when compared with control hearts (Figure 2B). In fact, the front end of coronary vessels in control hearts almost reached the apex and covered the majority of the ventricular region at E13.5 and E14.5, while coronary vessels in mKO hearts were restricted in the middle or near the base of the ventricles at the same stages (Figure 2A and Supplementary Figure 6A). Furthermore, the branching index, the total vessel length, and the density of coronary vessels were also significantly reduced in mKO hearts at E13.5 (Figure 2B). We further performed sectional immunostaining analysis to characterize the localization and the numbers of coronary vessels in both control and mKO hearts. Consistent with the results revealed by the whole-mount PECAM1 staining, coronary vessels in control hearts could be easily detected in the periphery of the myocardium from the base to the apex of the ventricle at both E13.5 and E14.5 (Figure 2C and Supplementary Figure 6B). However, coronary vessels could be observed only near the base of ventricles in mKO hearts at the same stages (Figure 2D and Supplementary Figure 6B). The statistical result also showed that the density of coronary vessels was significantly decreased in both left and right ventricles of mKO hearts (Figure 2E). Taken together, these results clearly demonstrated that deletion of SETD2 impaired the development of coronary vasculature in mouse hearts.

We also examined the effects of SETD2 deletion on the development of yolk sac vasculature and placenta. However, we did not observe a significant difference in vascular development between control and mKO yolk sacs at E14.5 (Supplementary Figure 7A). The placental histology and the thickness of placental labyrinth layer were also comparable between control and mKO mice at the same stage (Supplementary Figures 7B,C).

### SETD2 Regulates H3K36me3 and Downstream Gene Expression in Embryonic Mouse Hearts

**FIGURE 3:**
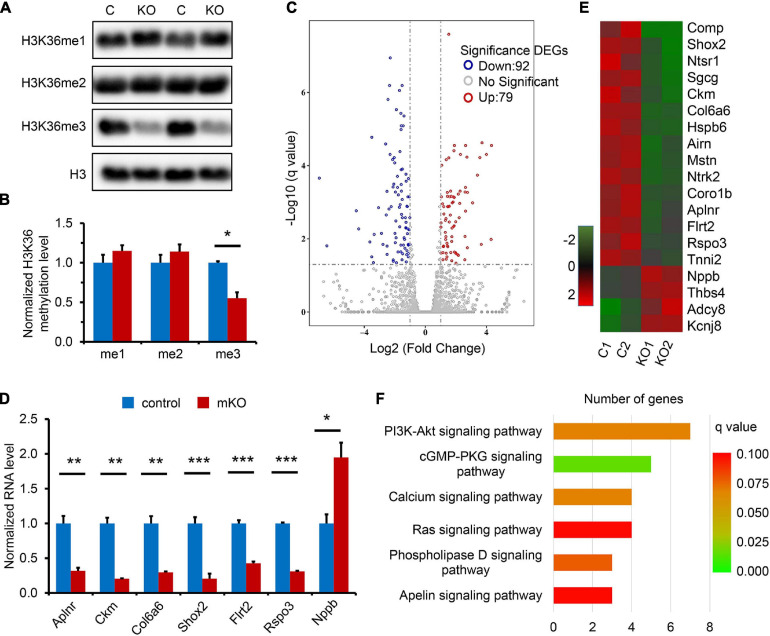
SETD2 regulates H3K36me3 level and downstream gene expression. **(A)** Western blotting of H3K36me1, H3K36me2, and H3K36me3 levels in control (C) and mKO (KO) hearts at E13.5. Histone 3 (H3) was used as the internal control. **(B)** Quantification of levels of H3K36me1, H3K36me2, and H3K36me3 normalized to Histone 3. *n* = 3 for each group. All data represent mean ± SEM. Significance was determined by two-tailed, unpaired Student’s *t* test. *p < 0.05 versus control. **(C)** Volcano plot of gene expression in control and mKO hearts at E12.5, indicating that 92 genes (blue circles) were downregulated and 79 genes (red circles) were upregulated in mKO hearts. Log2 fold change ≥ 1 and *q* value ≤ 0.05 are taken as the threshold. DEGs, differentially expressed genes. **(D)** RT-qPCR analysis of representative cardiac genes that are downregulated or upregulated by *Setd2* deletion. *n* = 5 for each group. All data represent mean ± SEM. Significance was determined by two-tailed, unpaired Student’s *t* test. *p < 0.05, **p < 0.01, ***p < 0.001 versus control. **(E)** Heatmap of the DEGs that are related to heart development in control and mKO hearts. Two independent experiments are noted as numbers 1 and 2. Color bar indicates relative expression levels. **(F)** KEGG analysis of the most altered pathways in mKO heart. The horizontal axis represents the number of genes. Color bar indicates *q* value.

We next sought to investigate the molecular changes underlying the regulation of SETD2 in coronary vascular development in mouse hearts. We measured H3K36me1, H3K36me2, and H3K36me3 in control and mKO hearts at E13.5 to determine the effects of SETD2 deletion on the methylation of H3K36. We found that only H3K36me3 was significantly decreased after deletion of SETD2 in hearts, whereas H3K36me1 and H3K36me2 were comparable between control and mKO hearts at this stage (Figures 3A,B), suggesting that SETD2 might only regulate the tri-methylation of H3K36 without affecting the mono-methylation and di-methylation of H3K36 in embryonic mouse hearts.

Functionally, both SETD2 and H3K36me3 have been demonstrated to activate mRNA transcription initiation and elongation, leading to increased gene transcription (Li et al., 2016); we therefore proposed that SETD2 might function through H3K36me3-mediated activation of key downstream genes. First, we performed qRT-PCR and measured the expression of *Notch*1 and Notch signaling-related genes including *Bmp10*, *Hey1, Hey2*, *Hes*1, *Hes6*, *Nrg1*, *Erbb2*, and *Erbb4* in control and mKO hearts at E13.5 but found that most of these genes were not significantly altered after SETD2 deletion, which is consistent with no apparent defects in ventricular compaction and cell proliferation observed in mKO hearts at this stage (Supplementary Figure 8). Next, we performed mRNA-seq to determine the transcriptional differences between mKO and control hearts at E12.5. At E12.5, no apparent morphological changes of hearts and embryos could be found in mKO mice (Figure 1A and Supplementary Figure 3B); we found that deletion of SETD2 had affected the cardiac transcriptome. As a result, a total of 171 genes were identified as being significantly altered (log2 fold > 1.0, q < 0.05), with 79 being upregulated and 92 being downregulated (Figure 3C). The Pearson correlation coefficient hierarchical cluster plot showed strong correlations within either control or mKO samples (both R^2 > 0.95; Supplementary Figure 9). Changes in gene expression were also confirmed by another transcriptome sequencing in two more pairs of control and mKO samples (Supplementary Figure 10). We also performed the qRT-PCR analysis to validate the changes of some cardiac genes between control and mKO embryonic hearts (Figure 3D). The Gene Ontology analysis revealed that 19 genes were proposed to be involved in cardiac developmental processes, 15 of which encode cardiac development regulators or important protein components of cardiomyocytes, such as genes that control muscle contraction: *Tnni2*, *Hspb6*, and *Ckm*; a gene that encodes the cardiac arrhythmia-associated transcription factor SHOX2; and another 4 genes involved in coronary vascular development: *Aplnr*, *Ntrk2*, *Flrt2* and *Rspo3* (Figure 3E). KEGG analysis showed that these genes were enriched in several important signaling pathways, including the PI3K-Akt, cGMP-PKG, calcium, and Ras signaling pathway, highlighting the potential role of these genes in heart development (Figure 3F).

### Rspo3 and Flrt2 Require SETD2 and H3K36me3 to Maintain Transcription in Hearts

**FIGURE 4:**
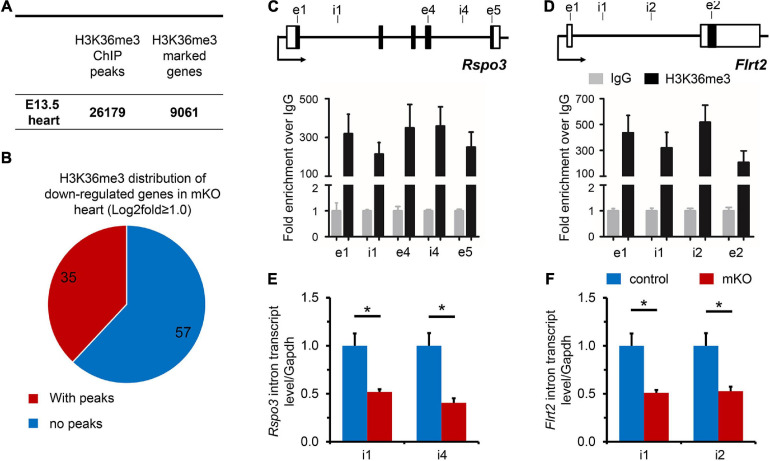
H3K36me3 is required for *Rspo3* and *Flrt2* transcription in the heart. **(A)** The numbers of H3K36me3 ChIP-seq peaks and H3K36me3 marked genes in E13.5 embryonic hearts. **(B)** Pie diagram illustration of H3K36me3 enrichment on downregulated genes in mKO hearts revealed by mRNA-seq. The numbers of genes were labeled in the graph. **(C,D)** ChIP-qPCR analysis of H3K36me3 occupancy to the locus of *Rspo3* and *Flrt2* genes, using IgG as the control. The locations of primers are indicated in the upper diagram. *n* = 3 for each group. Each bar represents the mean ± SEM. i, intron. e, exon. **(E,F)** qPCR analysis of the expression of *Rspo3* and *Flrt2* heterogeneous nuclear RNA introns in control and mKO hearts, using the primers indicated in the upper diagram of panels **(C,D)**, respectively. *n* = 5 for each group. All data represent mean ± SEM. Significance was determined by two-tailed, unpaired Student’s *t* test. *p < 0.05 versus control.

To uncover the distribution of H3K36me3 enrichment on the embryonic cardiac genome, ChIP-seq was then performed, and a total of 26,179 peaks (marked 9061 genes) were identified in E13.5 hearts, demonstrating a widespread H3K36me3 regulation in whole genomic regions, including introns, exons, and TTS, but rarely intergenic zones and promoters, where the occupancy of H3K36me3 peaks was extremely low (Figure 4A and Supplementary Figure 11A). Precious studies proposed that SETD2 and H3K36me3 were associated with actively transcribed genes possibly though stabilizing transcriptional elongation (Keogh et al., 2005), preventing erroneous transcription initiation events along the gene body (Neri et al., 2017), or establishing chromatin territories (Xu et al., 2019). We found that H3K36me3 was enriched in specific cardiovascular genes such as *Nkx2-5*, while H3K36me3 aggregation generally decreased in untranscribed genes and inter-genetic regions (Supplementary Figures 11B–D). We next focus on the downregulated genes observed in the mRNA-seq analysis. Among 92 downregulated genes in mKO hearts, 35 of total genes were observed with abundant H3K36me3 distribution (Figure 4B). Considering the distribution pattern of H3K36me3, a cross-examination of all these 35 genes in ChIP-seq with those 19 cardiac relative candidate genes observed was further analyzed and revealed that H3K36me3 was bound to *Rspo3* and *Flrt2* at gene body regions (Figures 4C,D and Supplementary Figure 11B). Intriguingly, both RSPO3 and FLRT2 are functionally required for normal cardiac development in mice. RSPO3 is an important downstream target of NKX2-5 (Cambier et al., 2014) and is primarily produced by cardiomyocytes (Da Silva et al., 2018). Furthermore, RSPO3 has been proposed to play an essential role in regulating coronary artery formation through activating Wnt/b-catenin signaling (Cambier et al., 2014; Da Silva et al., 2017), and deletion of *Rspo3* in mice has been shown to cause defective coronary stems and improper arterial tree formation (Da Silva et al., 2017, 2018). On the other hand, FLRT2, a fibronectin leucine-rich repeat transmembrane protein, is highly expressed in the epicardium and is required for normal cardiac morphogenesis (Muller et al., 2011). Deletion of *Flrt2* in mice disrupts epicardial integrity and causes embryonic lethality at around E12.5 in most mutant embryos (Muller et al., 2011). Therefore, we next investigated whether SETD2 could function through activation of *Rspo3* and *Flrt2*. We thus measured the expression of intron-containing nascent transcripts and found that the levels of either *Rspo3* or *Flrt2* nascent transcripts were significantly decreased in E13.5 mKO hearts compared with control hearts (Figures 4E,F), providing a direct evidence that deletion of SETD2 in embryonic hearts resulted in less H3K36me3 occupancy and decreased pre-mRNA transcription of *Rspo3* and *Flrt2* in mouse hearts. Although more studies should be performed in the future to determine whether restoring the expression of *Rspo3* or *Fltr2* could rescue cardiac abnormalities in SETD2 knockout mice, our results at least suggested that SETD2 is required for normal transcription of *Rspo3* and *Flrt2* during embryonic cardiac development, which might contribute to coronary vascular defects in SETD2 mutant hearts.

## Discussion

In our present study, we generated a mouse model with deletion of SETD2 by Mesp1-Cre and revealed an essential role of SETD2 in regulating coronary vascular formation and myocardium compaction during embryonic development. In adult hearts, the coronary arteries supply oxygen and nutrients to cardiomyocytes. Failure of coronary circulation may result in ischemic infarction and cardiac arrest, which is so far the leading cause of death worldwide. Therefore, an understanding of coronary vessel formation during embryonic development, their maintenance in adult hearts, and their remodeling under pathological conditions will be extremely important for developing new strategies to prevent or treat ischemic heart diseases (Hu and Kurpios, 2018; Lupu et al., 2020). Although the origin of coronary vessels during embryonic development is still under debate, recent studies using cell lineage tracing and single-cell transcriptome analysis provided convincing evidence that coronary vessels derive from endothelial sprouts of the sinus venosus with a small contribution from the endocardium lining the cardiac chambers (Red-Horse et al., 2010; Su et al., 2018). Interestingly, we found that loss of SETD2 did not block this initial step of coronary vessel development, evidenced by normal sprouting of endothelial cells from sinus venosus and formation of coronary vessel plexus at the base of mKO hearts at E12.5. However, deletion of SETD2 severely impaired the spreading/explanting of coronary vessels from the base toward the apex in embryonic hearts. Our results also suggested that formation of coronary vessels in left ventricles was more severely affected by loss of SETD2 than right ventricles in mouse hearts. However, the molecular mechanisms underlying the difference of coronary vessel formation between left and right ventricles of mKO hearts should be determined in the future.

We also observed a dramatic ventricular non-compaction phenotype in SETD2-mutant hearts. In general, non-compaction results from a failure of the final phase of cardiac development, the myocardial compaction process (Sedmera et al., 2000; Hussein et al., 2015). During embryonic development, cardiomyocyte precursors originate from the mesodermal layer and differentiate to the myocardium. Protrusions from the endocardial layer develop into myocardial trabeculations, which enable an increased surface-to-volume ratio and an increased myocardial mass before coronary arteries are formed (Bennett and Freudenberger, 2016; Finsterer et al., 2017). Subsequently, these trabeculations undergo a compaction process and the myocardium gradually compacts inward from the epicardium and from the base to the apex (Malla et al., 2009; Hussein et al., 2015). During compaction, proliferative activity is consistently higher within the compact myocardium, thus generating a gradient of decreasing proliferation of cardiomyocytes from the compact zone toward the trabecular zone (Pasumarthi and Field, 2002). Our results also confirmed a higher proliferative rate in the compact zone. However, we did not observe significant difference in cardiomyocytes proliferation between mKO and control hearts. On the other hand, coronary vasculature may also play a critical role in regulating cardiac development including ventricular compaction (Zhang et al., 2013; Finsterer et al., 2017; Wu, 2018). In fact, the development of myocardium and coronary vasculature is coupled at both molecular and cellular levels. Numerous studies have suggested that bone morphogenetic proteins, fibroblast growth factors, vascular endothelial growth factors, apelin, and angiopoietin 1 signaling may couple myocardial and coronary vascular development (Bhattacharya et al., 2006; Lupu et al., 2020). Our results demonstrated that defects of coronary vessel development arise as early as E12.5 in SETD2 knockout hearts, which precedes the appearance of abnormal myocardium development observed at E14.5 mKO embryos, implicating that coronary vascular abnormalities might be the primary cause of cardiac hypoplasia in mutant hearts. It is important to note that Mesp1-Cre may target multiple cardiovascular lineages including cardiomyocytes and endothelial cells (Saga et al., 1999; Yang et al., 2020). Therefore, cardiac abnormalities observed in mKO embryos might be due to cardiomyocytes, endothelial cells, or even in combination. In fact, our ongoing studies are now generating cardiac cell-specific and endothelial cell-specific SETD2 knockout mouse models to address this issue.

SETD2 has been recognized as the predominant methyltransferase in mammals that can tri-methylate histone H3 at lysine 36 (Sun et al., 2005). Consistently, our results demonstrated that deletion of SETD2 dramatically reduced the H3K36me3 level without affecting the levels of H3K36me1 and H3K36me2. Furthermore, we combined ChIP-seq and mRNA-seq techniques and identified *Rspo3* and *Flrt2* as representative genes that required SETD2 and H3K36me3 to maintain their transcription process. We also confirmed that both genes are bound with H3K36me3, and loss of SETD2 reduced pre-mRNA transcription of both genes. Intriguingly, both genes may play critical roles in regulating embryonic development and survival. In particular, RSPO3 is derived from cardiomyocytes and binds to LGR4 receptor and has been proposed to promote coronary endothelial stem cell proliferation in the developing heart (Cambier et al., 2014; Da Silva et al., 2017, 2018). Deletion of *Flrt2* in mice disrupts epicardial integrity and causes embryonic lethality at around E12.5 in most mutant embryos (Muller et al., 2011). These results at least implicated that reduced expression of *Rspo3* and *Flrt2* may contribute to coronary vascular defects in SETD2-mutant hearts. On the other hand, H3K36me3 has been correlated not only with the transcriptional activation and elongation but also with the regulation of DNA mismatch repair, homologous recombination, and alternative splicing (McDaniel and Strahl, 2017). In addition, SETD2 may also methylate non-histone targets including α-tubulin and signal transducer and transcription activator (STAT1) (Park et al., 2016; Chen et al., 2017). However, whether SETD2 regulates embryonic heart development via these mechanisms should be investigated in the future.

Although SETD2 has been shown to regulate numerous physiological processes, the role of SETD2 in cardiac development remains unknown. Therefore, we demonstrated for the first time, at least to our knowledge, that SETD2 and H3K36me3 are essential for embryonic cardiac development, mainly via regulating coronary vessel formation and myocardial compaction. These results also highlighted that epigenetic modulation may play a critical role in regulating cardiac morphogenesis and function and suggested that environmental factors affecting the expression of SETD2 or the level of H3K36me3 in cardiac tissues should be taken account for high risk for birth defects and prevented before and during pregnancy.

## Data Availability Statement

The original contributions presented in the study are publicly available. This data can be found here: Bio Project, https://dataview.ncbi.nlm.nih.gov/object/PRJNA692266reviewer=eotm6c6bi48sthpvavtol6idug, accession number: PRJNA692266.

## Ethics Statement

All mice were housed under a 12-h day/night cycle at a temperature of 25°C. All animal care and experiments were conducted in accordance with the guidelines established by the Animal Care and Use Committee (IACUC) at Peking University Shenzhen Graduate School (Shenzhen, China) and approved by the IACUC (Approval #AP0017). A periodic review of procedures was performed, and amendments were made as needed.

## Author Contributions

FC, JC, HW, HT, LH, and XW performed the experiments. LL, KO, and ZH designed the research. SW, JL, and LH provided the material. XF, LL, KO, and ZH wrote the manuscript. All authors contributed to the article and approved the submitted version.

## Conflict of Interest

The authors declare that the research was conducted in the absence of any commercial or financial relationships that could be construed as a potential conflict of interest.
