# Supplementary material for: Histone Lysine Methyltransferase SETD2 Regulates Coronary Vascular Development in Embryonic Mouse Hearts
Source: Front Cell Dev Biol. 2021 Apr 9;9:651655. doi: 10.3389/fcell.2021.651655 (PMC8063616; doi:10.3389/fcell.2021.651655)
Supplement: Supplementary file 1 [file Table_1.DOCX]

**Supplemental Table 1. Sequences of primers used for qRT-PCR.**

| Gene | Forward primer (5’→3’) | Reverse primer (5’→3’) |
| --- | --- | --- |
| *Setd2* | GTTCCTCTCGATGTCCAAATGG | GCAATTCCCTTTTTGAGTGGC |
| *Ckm* | GGACCCCAACTATGTGCTGA | GTACTTGCCCTTGAACTCGC |
| *Rspo3* | AAGGGTTAGAAGCCAACAATCATACTAT | AGGATGCTGTAGTATATCTCGGACC |
| *Flrt2* | CTGGATGCGTTCAACTACCG | GAGCAAGACAACGAGCACAA |
| *Shox2* | GCGAAGTCGGACCAATTTTA | AACTGGCTAGCGGCTCCTAT |
| *Aplnr* | GGTGGCCACTTCAAACTCAG | GGGCAAAGGTCACTACAAGC |
| *Col6a6* | CTGACCAATGGCATGTCTCG | TGTCTCTGCAGGCTTCTTCA |
| *Nppb* | AAGCTGCTGGAGCTGATAAGA | GTTACAGCCCAAACGACTGAC |
| *Gapdh* | ATGACTCCACTCACGGCAAAT | TCCCATTCTCGGCCTTGAC |
| *Tbx20* | TATTCAGCATACTCCTAC | GTTAGTCTTGTCAATACG |
| *Hey1* | CAGTGCCTTTGAGAAGCAGG | CAGATAACGGGCAACTTCGG |
| *Hey2* | AAGCGCCCTTGTGAGGAAAC | GGTAGTTGTCGGTGAATTGGAC |
| *Notch1* | AGCCTCTCCACCAATACCTT | GGCTGGAGCTGTAAGTTCT |
| *Bmp10* | ATGGGGTCTCTGGTTCTGC | CAATACCATCTTGCTCCGTGAA |
| *Cx40* | AGCAACATACCAGATAGA | GCAGATTACTTGATGAATAC |
| *Nrg1* | ATGGAGATTTATCCCCCAGACA | GTTGAGGCACCCTCTGAGAC |
| *ErbB2* | ACCGACATGAAGTTGCGACTC | AGGTAAGCTCCAAATTGCCCT |
| *ErbB4* | GTGCTATGGACCCTACGTTAGT | TCATTGAAGTTCATGCAGGCAA |
| *Hes1* | TCATGGAGAAGAGGCGAAGG | GTCACCTCGTTCATGCACTC |
| *Hes6* | GCTACATCCAGTGCATGCAT | GACAATGGGGATTCTGGGGA |

**Supplemental Table 2. Sequences of primers used for ChIP-qPCR.**

| Gene | Forward primer (5’→3’) | Reverse primer (5’→3’) |
| --- | --- | --- |
| *Rspo3-exon1* | AGAACTGAAGGACATGGCCA | GGTTTGGTGGTTTCTGTCCC |
| *Rspo3-intron1* | TAATGACAGCTGGCCAGTGA | CCTGCAGTTGGTATGGGGAA |
| *Rspo3-exon4* | GGGTCCGAGATATACTACAGCA | TCTCCCTTTGAACACTTCTTTCT |
| *Rspo3-exon5* | TTAGGATTGCTTCGGTTGCC | CACGGTTCTGGAAAGCACAA |
| *Rspo3-intron4* | CTCCTGCTGTTAATCTCCTGG | GGCCAGTTTGCAACCTAATGT |
| *Flrt2-intron1* | TGAGTGCAGCAAAGGATATGTG | TTTGGTCGGCAAGAAGAGAC |
| *Flrt2-intron2* | ATCTCGTGATACCTCCGCAG | TGCAGGGACATCACCACTAA |
| *Flrt2-exon1* | GCTGAAGTACTGTACGGGGT | GTTCTGACAGCCTCAACAGC |
| *Flrt2-exon2* | GCGCTCTTATCTGGTCCTCT | TCGTTGAGAACTGCCAGTCT |
