# Supplementary material for: Histone Lysine Methyltransferase SETD2 Regulates Coronary Vascular Development in Embryonic Mouse Hearts
Source: Front Cell Dev Biol. 2021 Apr 9;9:651655. doi: 10.3389/fcell.2021.651655 (PMC8063616; doi:10.3389/fcell.2021.651655)
Supplement: Supplementary file 2 [file Data_Sheet_1.PDF]

Supplemental Figures and legends

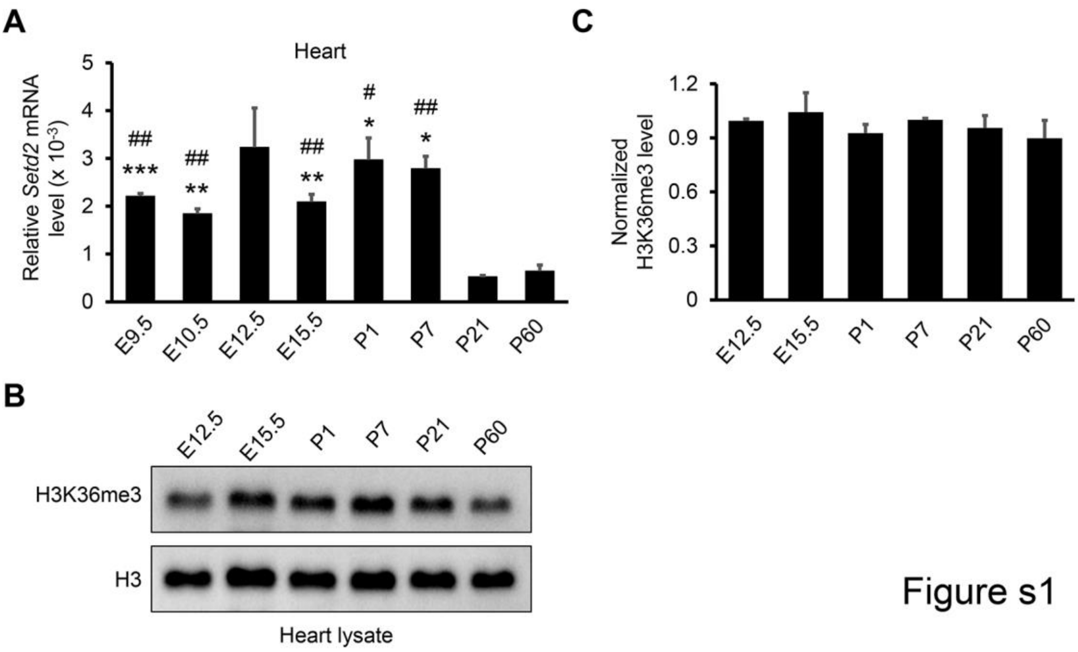

Supplemental Figure 1. Temporal expression of *Setd2* and H3K36me3 in heart development. (**A**) qRT-PCR analysis of *Setd2* mRNA levels in hearts from the embryonic day 9.5 (E9.5) to weaning (P21) and P60. n = 3 for each group. All data represent mean ± SEM. Significance was determined by two-tailed, unpaired Student's t test. *p < 0.05, **p < 0.01, ***p < 0.001 versus P21; $^{\#}$p < 0.05, $^{\#\#}$p < 0.01, $^{\#\#\#}$p < 0.001 versus P60. (**B**) Western blot analysis of H3K36me3 levels in embryonic and postnatal mouse hearts. Histone 3 (H3) was used as the internal control. (**C**) Quantification of the levels of H3K36me3 normalized to H3 from E12.5 to adult mouse hearts. n = 3 for each group. All data represent mean ± SEM. Significance was determined by two-tailed, unpaired Student's t test.

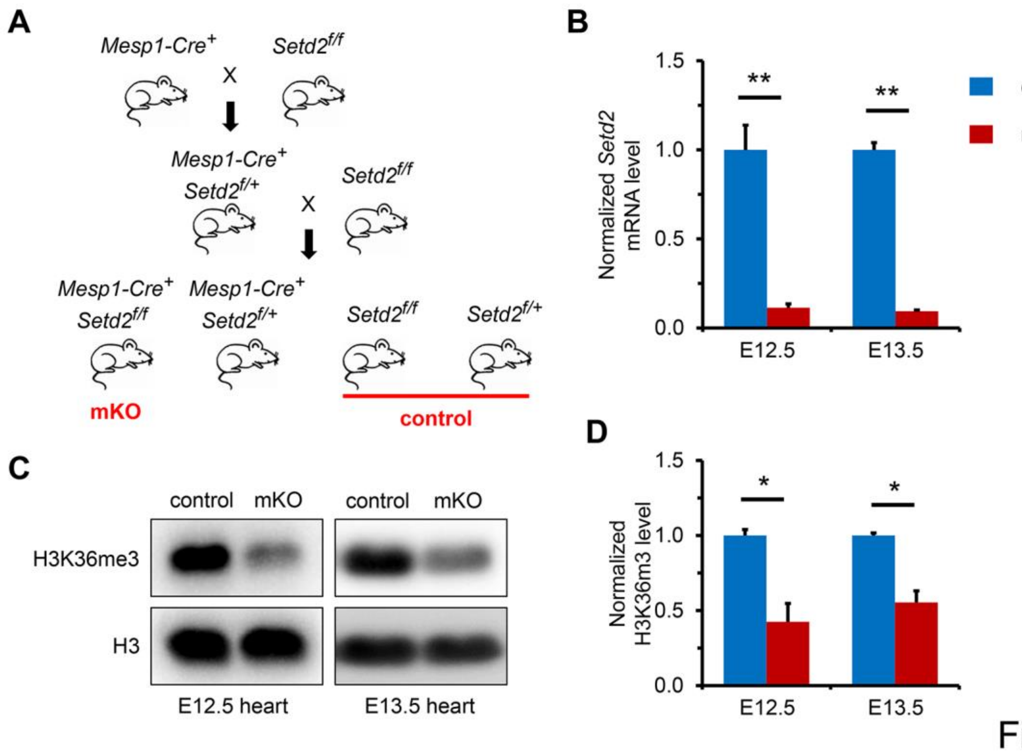

Figure s2

Supplemental Figure 2. Generation and characterization of cardiac progenitor-specific *Setd2* knockout mice. (**A**) Schematic diagram of the breeding strategy to generate cardiac progenitor-specific *Setd2* knockout mice. *Mesp1-Cre*$^{+}$*Setd2*$^{f/+}$ mice were first generated and then backcrossed with *Setd2*$^{f/f}$ mice to generate *Mesp1-Cre*$^{+}$*Setd2*$^{f/f}$ (mKO) mice and the littermate *Mesp1-Cre*$^{-}$*Setd2*$^{f/+}$ and *Mesp1-Cre*$^{-}$ *Setd2*$^{f/f}$ mice were used as control. (**B**) qRT-PCR analysis of *Setd2* mRNA expression in control and mKO hearts at E12.5 and E13.5, respectively. n = 4 for each group. All data represent mean ± SEM. Significance was determined by two-tailed, unpaired Student's t test. **$p < 0.01$ versus control. (**C**) Western blot analysis of H3K36me3 levels in control and mKO hearts at E12.5 and E13.5, respectively. Histone 3 (H3) was used as the internal control. (**D**) Quantification of the levels ofH3K36me3 normalized to H3. n =3 for each group. All data represent mean ± SEM. Significance was determined by two-tailed, unpaired Student's t test. *$p < 0.05$ versus control.

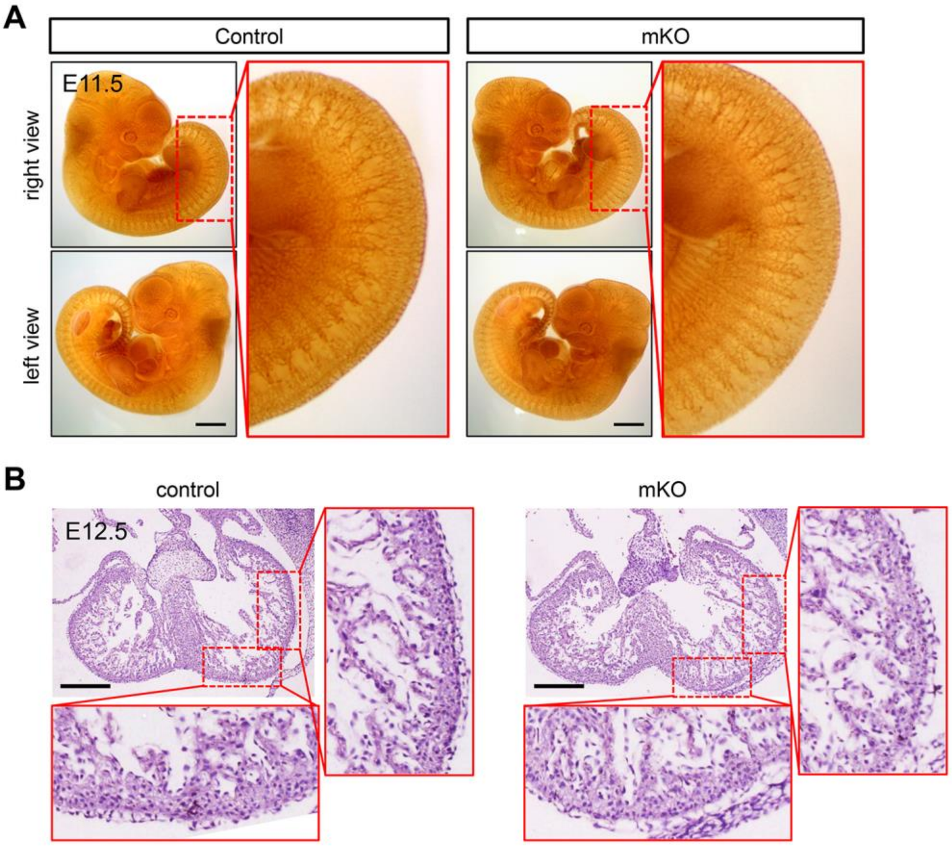

Supplemental Figure 3. Histologic analysis control and mKO embryos at E11.5 and E12.5. (**A**) Representative images of whole mount PECAM staining of E11.5 control and mKO embryos. Scale bar, 1mm. (**B**) Representative H&E stained sections of control and mKO embryonic hearts at E12.5. Scale bar, 500 μm.

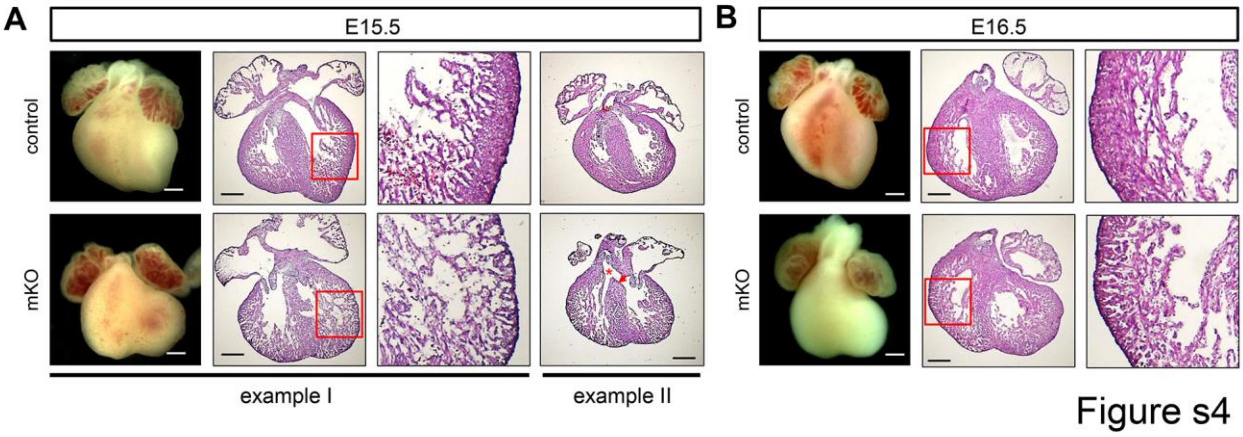

Supplemental Figure 4. Deletion of SETD2 leads to cardiac structural defects. Morphological and

histologic analysis were performed in control and mKO hearts at E15.5 (**A**) and E16.5 (**B**). Please note that control hearts developed densely and compact myocardium layers at both E15.5 and E16.5, while only a thin layer of compact zone was observed in mKO hearts at the same stages. Please also note that ventricular septal defect (red arrow) and double outlet right ventricle (red star) were observed in example II of mKO embryos at E15.5. Boxed areas are magnified on the right. Scale bar, 300μm.

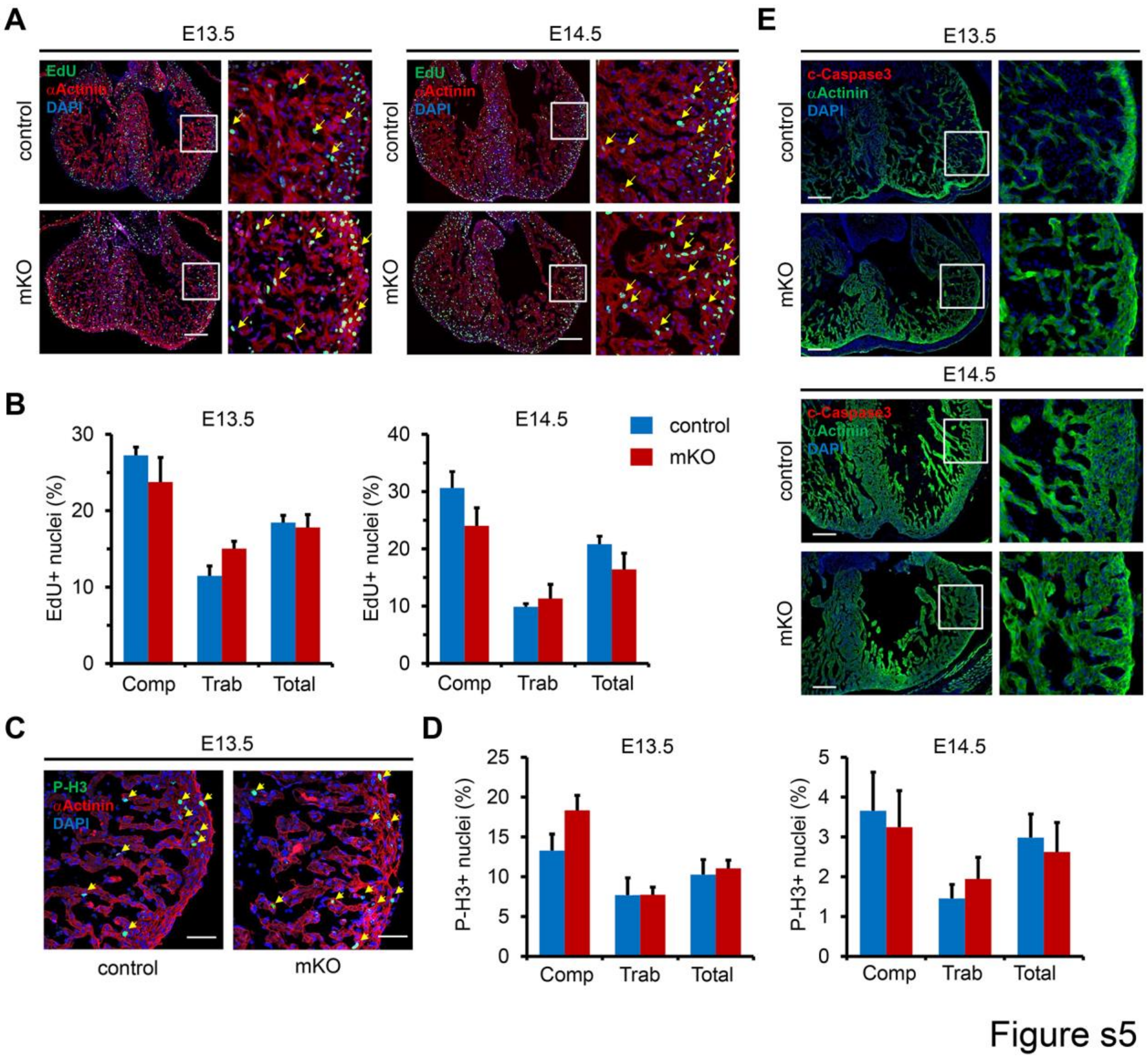

Supplemental Figure 5. Loss of SETD2 has no major effects on cardiac cell proliferation and apoptosis. (**A**) Cell proliferation was analyzed by immunostaining of incorporated EdU in control and mKO hearts at E13.5 and E14.5, respectively, with cardiac cells co-stained with α-actinin. Boxed areas are magnified on the right. Arrows indicate EdU-stained nucleus. Scale bar, 500 μm. (**B**) Quantification of

cardiomyocyte proliferation in the compact zone (comp), trabecular (trab), and whole heart (total). Mitotic indexes were calculated by dividing the number of EdU-positive nuclei by the total number of nuclei in the whole visual area. n = 4 for each group at E13.5, n = 3 for each group at E14.5. All data represent mean ± SEM. Significance was determined by two-tailed, unpaired Student's t test. (**C**) Representative pictures of immunofluorescence staining of phospho-Histone 3 (P-H3) in control and mKO embryonic hearts at E13.5, in which cardiomyocytes co-stained with α-actinin. Scale bar, 100 μm. (**D**) Quantification of cardiomyocyte proliferation in the compact zone (comp), trabecular (trab), and whole heart (total). n = 3 for each group. All data represent mean ± SEM. Significance was determined by two-tailed, unpaired Student's t test. (**E**) Cell apoptosis was assessed by immunostaining of anti-cleaved Caspase 3 (c-Caspase 3) in control and mKO hearts at E13.5 and E14.5, respectively, in which cardiomyocytes co-stained with α-actinin. Please note that c-Caspase 3 positive nucleus could be rarely observed in both control and mKO hearts. Scale bar, 500 μm.

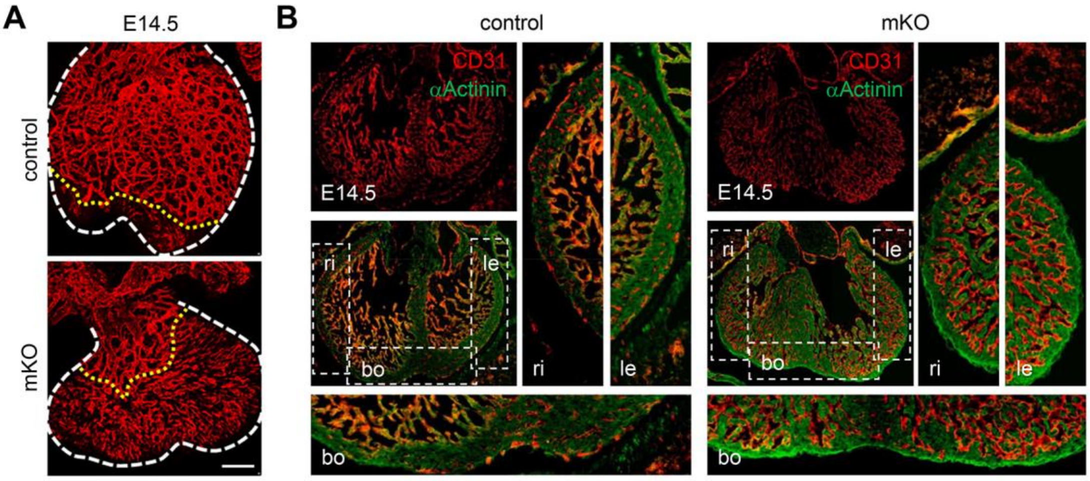

Figure s6

Supplemental Figure 6. Assessment of coronary vascular development in control and mKO hearts at E14.5. (**A**) Whole mount PECAM immunostaining of embryonic hearts at E14.5. Please note that white dotted line indicates the border of heart and yellow dotted line indicates the leading tips of coronary vessels. Scale bar, 500μm. (**B**) Immunostaining analysis of coronary vessel's distribution in control and mKO hearts at E14.5. Heart sections were prepared and stained with anti-α-Actinin for cardiomyocytes and anti-CD31 for endothelial cells, respectively. Please note that coronary vessels could be easily observed in left ventricle, right ventricle and the bottom area of control heart, while only very few coronary vessels could

be seen in the base of right ventricle in mKO heart. Scale bar, 40 μm. Ri, right ventricle. Le, left ventricle. Bo, bottom.

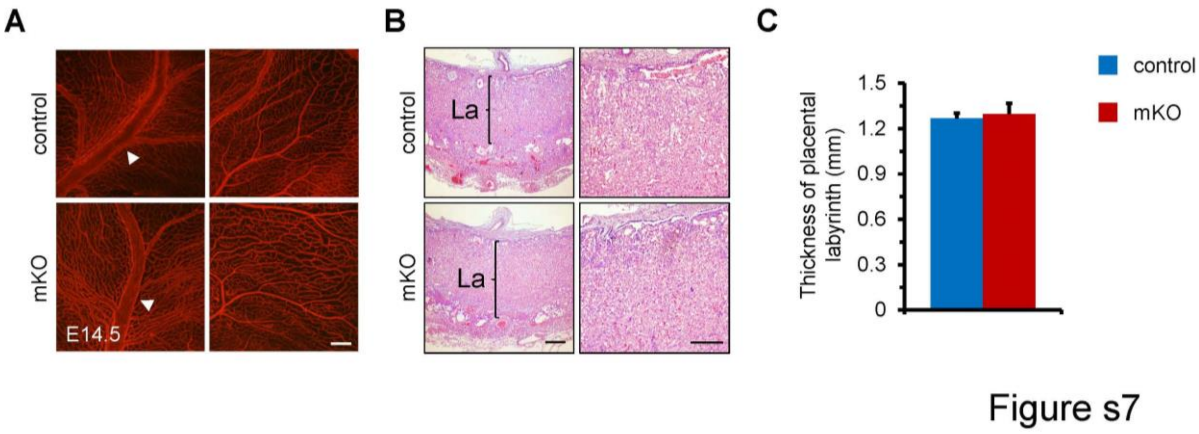

Supplemental Figure 7. Loss of SETD2 has no major influence on formation of yolk sac vasculature and placental development. (**A**) Whole mount PECAM immunostaining of yolk sac in control and mKO embryos at E14.5. Scale bar, 400μm. White arrowheads indicate the arteries in yolk sac. (**B**) Representative H&E stained sections of control and mKO placentas at E14.5. La, labyrinth. Scale bar, 80μm. (**C**) Quantification of thickness of the labyrinth in control and mKO placentas at E14.5. n = 5 for each group. All data represent mean ± SEM. Significance was determined by two-tailed, unpaired Student's t test.

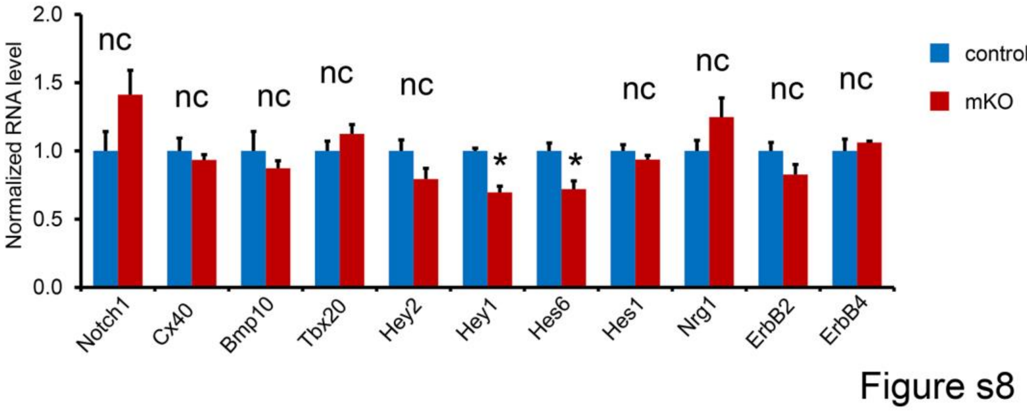

Supplemental Figure 8. mRNA levels of Notch1 and Notch signaling related genes. qRT-PCR analysis was performed to measure expression of each listed gene in control and mKO hearts at E13.5. n = 3 for each group. All data represent mean ± SEM. Significance was determined by two-tailed, unpaired

Student’s t test. *$p < 0.05$ versus control.

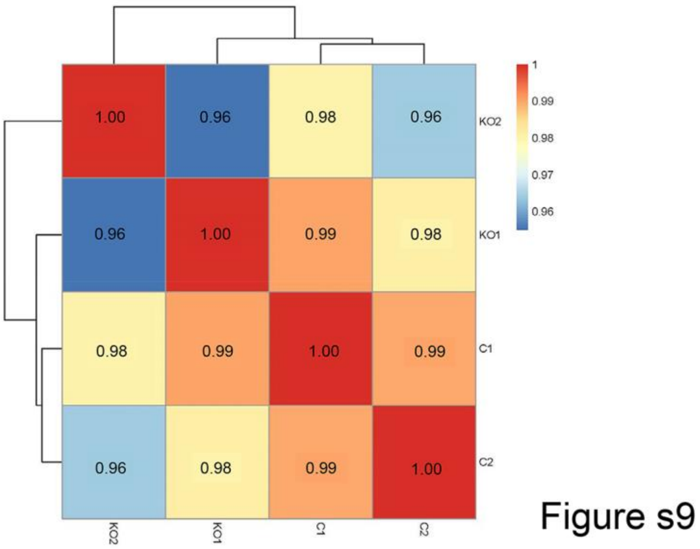

Supplemental Figure 9. Pearson correlation Coefficient hierarchical cluster plot within samples. The Pearson correlation Coefficient (R^2) of each group of samples was shown.

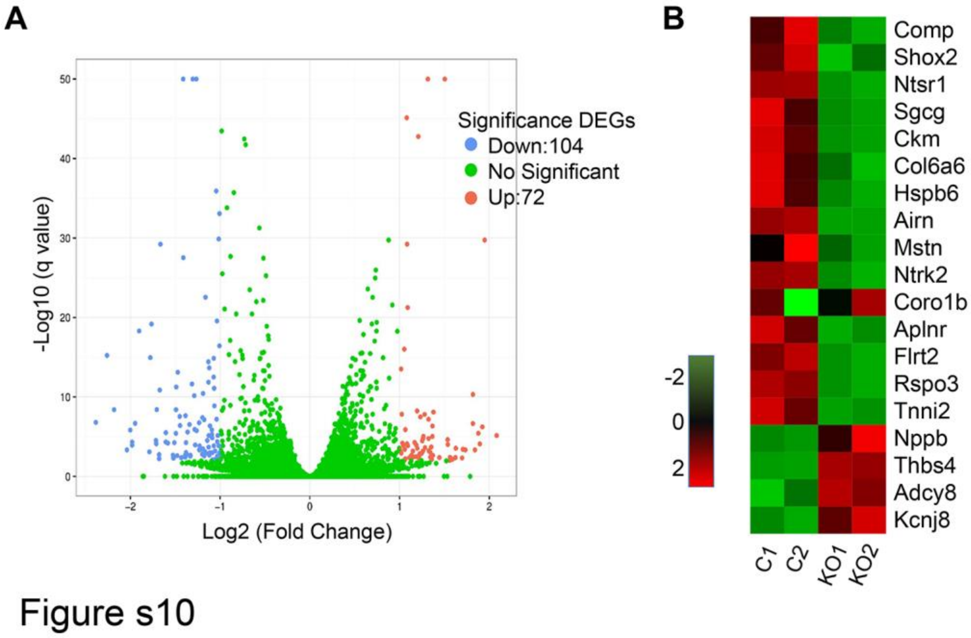

Supplemental Figure 10. Differentially expressed genes identified in mKO hearts from another

RNA-seq analysis. (**A**) Volcano Plot reveals 104 down-regulated genes (blue dots) and 72 up-regulated genes (red dots) in mKO versus control hearts determined by RNA-seq. Log2 fold change ≥1, q value ≤0.05 are taken as the threshold. DEGs, differentially expressed genes. (**B**) Altered expression of genes related to heart development in control and mKO hearts. Two independent experiments are noted as numbers 1 and 2. Color bar indicates relative expression levels.

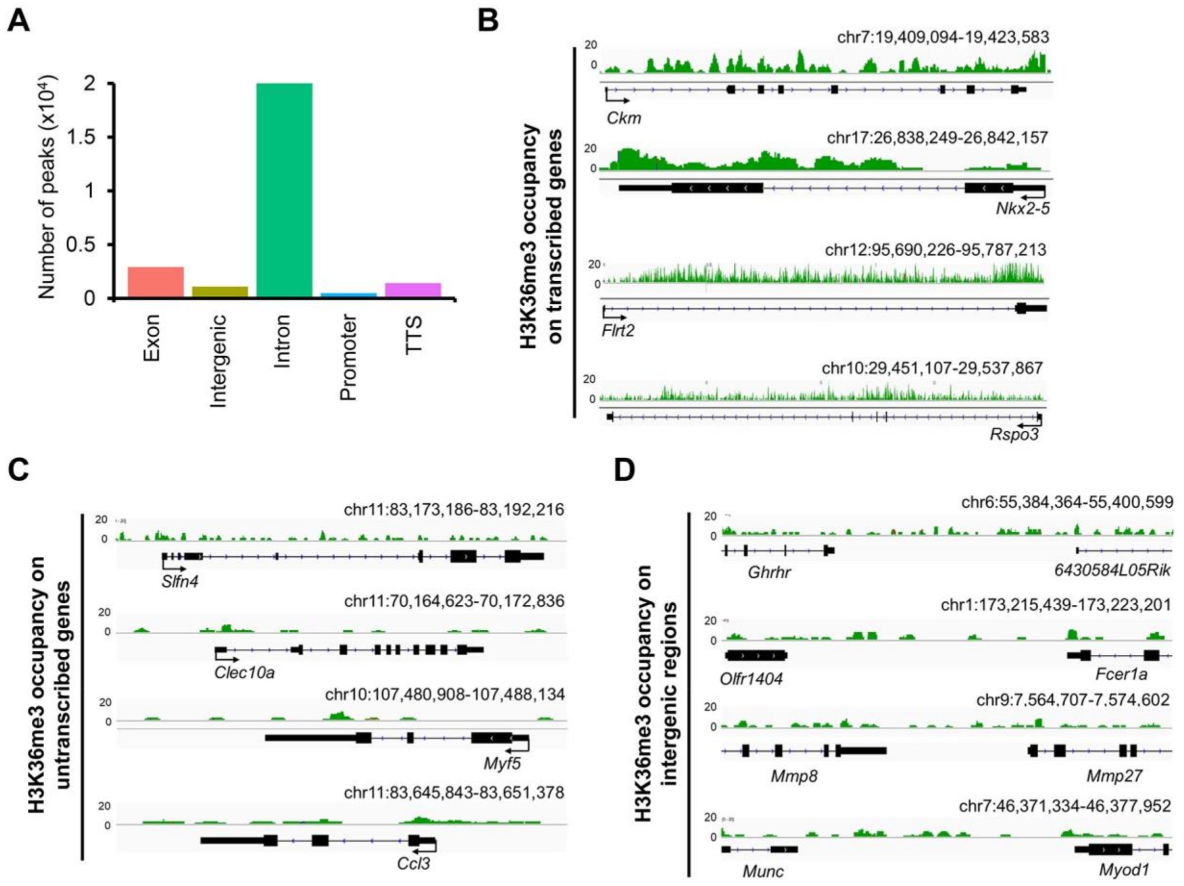

Figure s11

Supplemental Figure 11. H3K36me3 distribution on genomic elements and specific genes. (**A**) A widespread H3K36me3 distribution in whole genomic regions, including introns, exons, and TTS, but rarely intergenic zones and promoters, where the occupancy of H3K36me3 peaks were extremely low. TTS, transcription terminal site. (**B-D**) Profiles of H3K36me3 occupancy to transcribed genes (**B**) including *Ckm, Nkx2-5, Flrt2* and *Rspo3*, untranscribed genes (**C**), and intergenic regions (**D**) in embryonic hearts.
